# Laser Superficial Fusion of Gold Nanoparticles with PEEK Polymer for Cardiovascular Application

**DOI:** 10.3390/ma14040971

**Published:** 2021-02-18

**Authors:** Oktawian Bialas, Mateusz Lis, Anna Woźniak, Marcin Adamiak

**Affiliations:** Department of Materials Engineering and Biomaterials, Faculty of Mechanical Engineering, Silesian University of Technology, Konarskiego 18A Street, 44-100 Gliwice, Poland; mateusz.lis@polsl.pl (M.L.); anna.wozniak@polsl.pl (A.W.); marcin.adamiak@polsl.pl (M.A.)

**Keywords:** laser texturing, gold coating, PEEK treatment, cardiac application, cardiovascular supply, biomaterials

## Abstract

This paper analyses the possibility of obtaining surface-infused nano gold particles with the polyether ether ketone (PEEK) using picosecond laser treatment. To fuse particles into polymer, the raw surface of PEEK was sputtered with 99.99% Au and micromachined by an A-355 laser device for gold particle size reduction. Biomimetic pattern and parameters optimization were key properties of the design for biomedical application. The structures were investigated by employing surface topography in the presence of micron and sub-micron features. The energy of the laser beam stating the presence of polymer bond thermalisation with remelting due to high temperature was also taken into the account. The process was suited to avoid intensive surface modification that could compromise the mechanical properties of fragile cardiovascular devices. The initial material analysis was conducted by power–depth dependence using confocal microscopy. The evaluation of gold particle size reduction was performed with scanning electron microscopy (SEM), secondary electron (SE) and quadrant backscatter electron detector (QBSD) and energy dispersive spectroscopy (EDS) analysis. The visibility of the constituted coating was checked by a commercial grade X-ray that is commonly used in hospitals. Attempts to reduce deposited gold coating to the size of Au nanoparticles (Au NPs) and to fuse them into the groove using a laser beam have been successfully completed. The relationship between the laser power and the characteristics of the particles remaining in the laser irradiation area has been established. A significant increase in quantity was achieved using laser power with a minimum power of 15 mW. The obtained results allowed for the continuation of the pilot study for augmented research and material properties analysis.

## 1. Introduction

Laser texturing, in the form of periodic surface microstructures such as grooves, dimples, squares, etc., are mostly used to promote osseointegration in dental and orthopaedic implants applications [1,2,3]. Beside the long term osseointegration and dental/orthopaedic applications, there are also areas that are lifesaving. According to the World Health Organisation (WHO), the main cause of death is cardiovascular system failure [4]. That statement imposes a necessity of further improvement of the materials used for cardiac application to lower the re-operation and death rates, especially in short term after implantation of a foreign body into the system. Surface structuring through the use of a monochromatic light is a very beneficial method and is commonly studied for that purpose, being highly precise and flexible in terms of creating different possibilities to enhance surface morphology and chemistry [5,6,7]. Cell adhesion and proliferation are typically accomplished by all surface forms of structures, such as pits and posts, which have been explored on the surface of various materials such as polymers and metals. Increased adherence, density, monolayer formation, and endothelial cell proliferation have been documented [2,8,9]. Beside the mentioned advantages, it is important to create pattern that allows good endothelial cell alignment and provides no opportunity for factor XII to be activated during blood flow, which initiates the whole cascade responsible for inflammatory, thrombosis and clot formation of the blood [10,11,12]. The following article proposes laser beam micromachining as the most precise way to construct biomimetic micropatterns changing the surface properties. The knowledge suggests that for a new generation of (delicate and thin) cardiac devices, the most desirable depth for the surface modification should not exceed 5 µm [2]. Nevertheless, it is well known that modification through thin atomic layer deposition (ALD) affects the surface properties of the medical materials [13].

The current study focuses on laser beam material modification by deposition of Au particles and reduces its size to nanoscale by so-called top-down methods [14]. The modern material, polyether ether ketone (PEEK) tends to be chemically stable and has the potential to be applied as an alternative to titanium alloys conventionally used for implantation. Besides that, PEEK demonstrates a high resistance to gamma and electron beam radiation, that makes it consequently not considered as a source of secondary radiation after gamma sterilization. Li et al. suggest that free radicals produced by irradiation at 600 kGray have a lifetime not exceeding 20 min [15]. Furthermore, PEEK micromachined by a 355nm laser beam wavelength by causing only small thermal affection of the surface can improve cell adhesion and provide better conditions for organic tissue [16]. The range of the usability of PEEK has been described in a wide range of applications. Implants made from PEEK were divided in the clinical classification in five main groups: for bone replacement—maxilla-facial and cranial implants; for spine surgery—spinal cages; for orthopaedic surgery; for tooth replacement—dental implants; and for cardiac surgery—intracardiac pump and heart valves [17]. In the last decades, polymeric materials have caught wide attention in research as a metal-based implant replacement, as it has a greater affinity for organic tissue properties [18,19,20,21]. In the course of this study, the material was enriched in gold nanoparticles as a 200 nm thick layer coating selectively ablated/melted on the material surface. The gold nanoparticles are widely used as markers in X-ray, computer tomography (CT), magnetic resonance imaging (MRI), and positron emission tomography (PET) scans, etc., [22] and are desired to be applied in this case to evaluate the polymeric implant position after implantation and to control material degradation during the long-lasting perspective, as gold is considered to be biocompatible [23,24]. It is important, especially for cardiac devices, whose proper functionality depend on correct anatomic positioning. Chest radiographs are the initial modality to evaluate the device location and its integrity after implantation and for diagnosis of complications and malfunction. [25,26,27]. Antibacterial properties of gold were also desired as a potential lowering of implant failure in the early stages after operation time due to the reluctance of bacteria to settle on the surface [28].

## 2. Materials and Methods

Samples from polyether ether ketone (PEEK) were delivered as ø32 rods (Mitsubishi Chemical Advanced Materials, Lenzburg, Switzerland). The material is characterised by properties presented in Table 1.

The rod was cut into 7 mm-thick slices and prepared by grinding to prepare the surface for all tested variants (Table 2). Uniform flattening and surface preparation are necessary to conduct a comparative analysis [31]. The mechanical finishing process was performed using the grinding–polishing machine, TERGAMIN-30 (Struers, Willich, Germany). Each sample was mechanically grindined with paper grain-size gradation, sequentially 220, 800, 1200 grid/mm^2^ in time t = 4 min per each gradient, and mechanically polished by polishing wheels, with a gradation of 9, 6, 3, and 1 µm.

### 2.1. Sample Preparation

**Figure 1:**
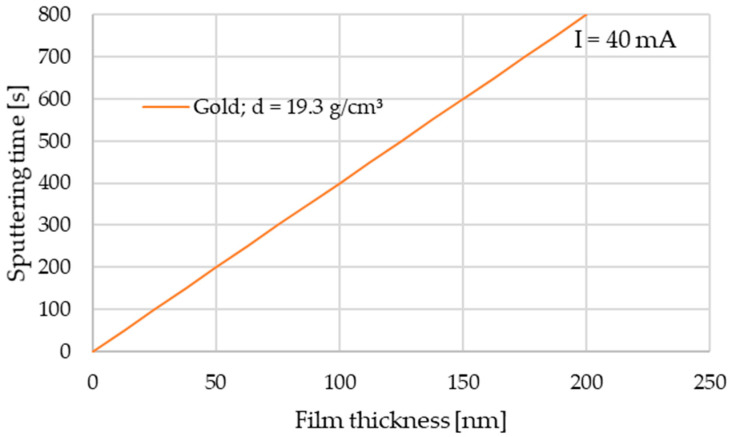
Sputtering time diagram [32].

Gold sputtering was performed using a Leica EM SCD050 device with a 99.99% Au target. The current parameter was set to 40 mA, and time was set to 800 s following the sputtering time diagram (Figure 1) for gold (d = 19.3 g/cm^3^) according to user’s manual [32].

**Figure 2:**
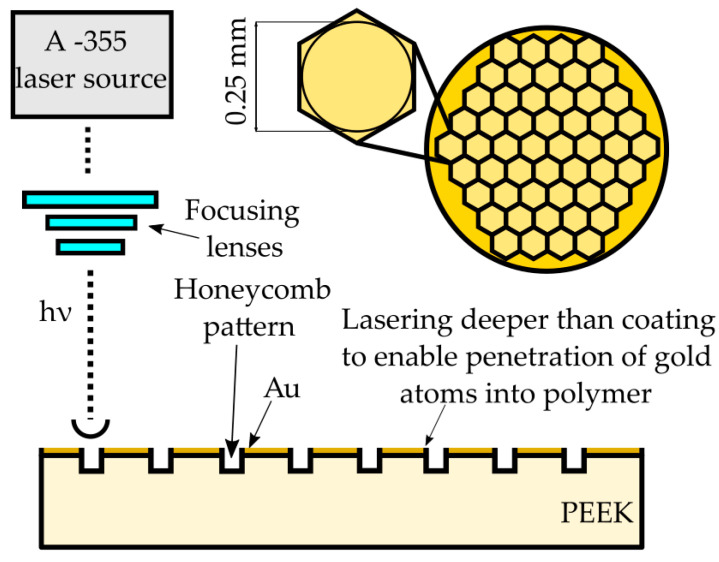
Laser micromachining scheme. PEEK: polyether ether ketone.

Samples were subjected to laser treatment (Table 3). The experiment was accomplished using an A-355 Laser Micromachining system (Oxford Lasers, Didcot, UK) based on a 355 nm wavelength diode-pumped solid-state picosecond laser. The system provides high energy density which allows one to ablate/melt the material. The maximum pulse energy for the A-355 picosecond Laser Micromachining system is 0.2 mJ with an average power of 23 mW, and the pulse duration is about 6 ps. The path of laser texturing was either spot created by a single laser pulse irradiation (at a frequency of 10 Hz and a speed of 1 mm/s) or regular hexagonal honeycomb pattern (at a frequency of 400 Hz and a speed of 10 mm/s) as a proposition for future mechanical properties studies with the 0.25 mm diameter of the circle inscribed (Figure 2). The laser pattern was set to keep the ratio of laser treated to non-laser treated surface close to 25%.

### 2.2. Microscopic Observations

High resolution scanning electron microscopy (HRSEM) was used to assess the stage of the melting/evaporating ratio. The equipment—Supra 35 (Zeiss, Oberkochen, Germany)—included EDS spectrum analysis, which allowed one to determine the surface texture and qualitative chemical composition. The tests were performed on the material covered with a layer of gold. The study was conducted in selected micro-areas to determine the fusion of gold into the polymer after laser treatment. The samples were also observed using a confocal microscopy system, LSM Exciter 5, supplied by Zeiss (Zeiss, Wetzlar, Germany). The system was equipped with a 405 nm diode laser with 25 mW of power to achieve the most relevant optical resolution. Every sample was analysed using the z-stack mode to investigate the selected structure of the laser groove decomposition and gained depth. After scanning, a 3D image was obtained, consisting of images of planes scanned successively. 

### 2.3. Medical X-ray Photo Imaging

To justify the purpose of gold sputtering as a marker agent, the X-ray photos were taken. The experiment was accomplished using a high frequency technology intra-oral X-ray system Trophy Elitys (Trophy Radiologie, Croissy–Beaubourg, France). For an exposure with a strong contrast of the obtained image, the following parameters were chosen: voltage: 60 kV, current: 7 mA, exposure time: 0.63 s.

## 3. Results

**Figure 3:**
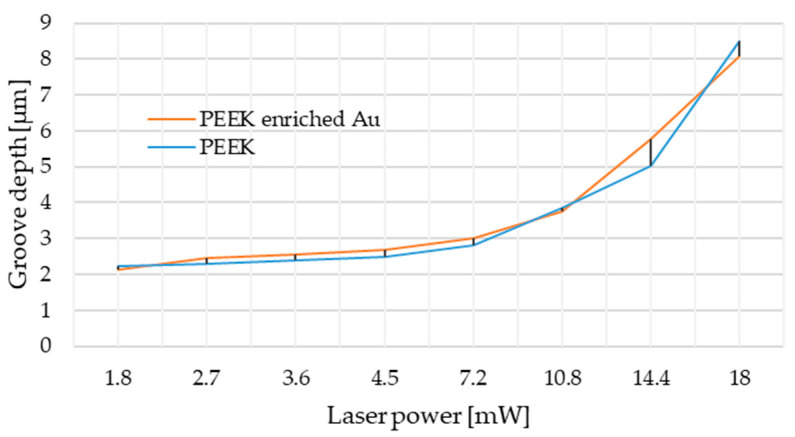
Power depth laser diagram based on confocal system analysis.

In the first stage of material testing, it was planned to create a plot of laser power versus depth at the frequency of 400 Hz and the cutting speed of 1 mm/s (Figure 3). Based on the obtained results, the best parameters were chosen to perform experiment, taking into account the initial objectives and absorbance of the laser beam by the tested matter. The obtained results allowed one to conclude that considerate laser powers should be from the range between 4.5 mW and 18 mW because of the rapid income of depth in that region (from 2.2 µm for 4.5 mW to over 8 µm for 18 mW). Due to the enforced change of frequency parameters which caused higher nanoparticles lasting, the single laser irradiation approach was applied; the authors chose 4.5 mW as the minimum, and 15 mW and 16 mW as the optimum.

**Figure 4:**
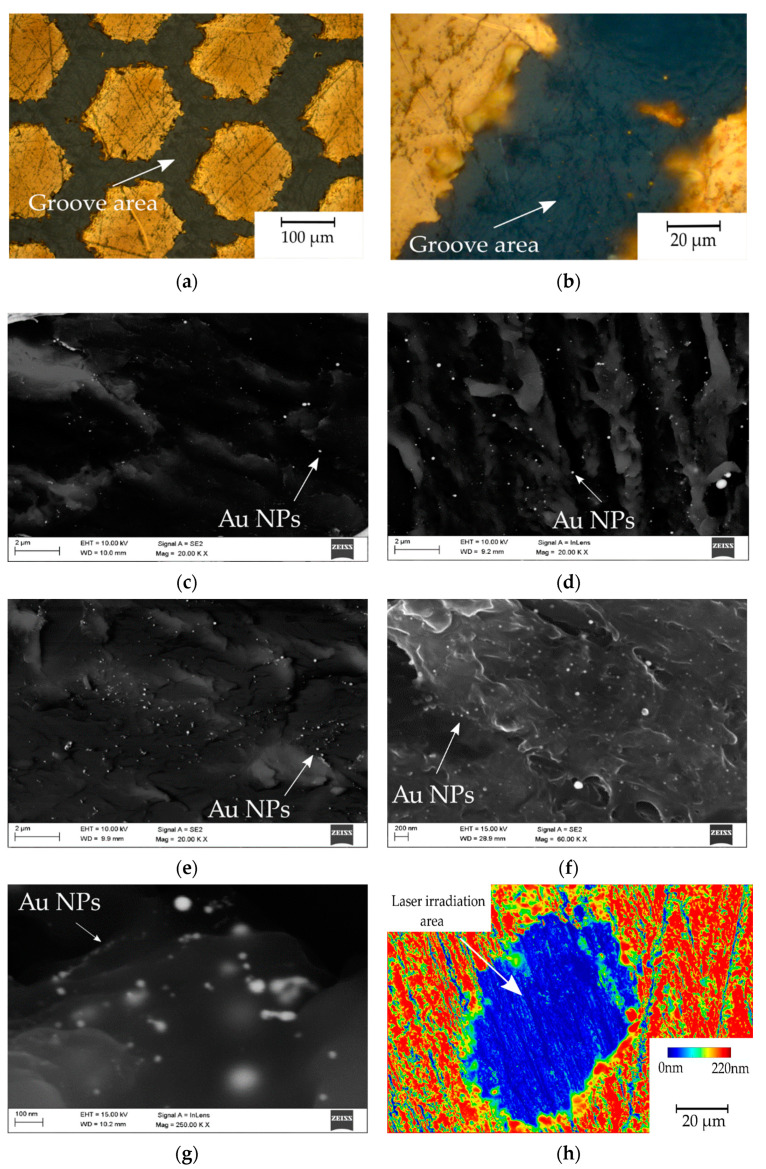
Gold particles inside the groove area: (**a**) 15 mW—light microscopy; (**b**) 15mW groove—light microscopy (**c**) groove 4.5 mW, SEM, SE+QBSD; (**d**) groove 15 mW, SEM, secondary electron and quantitative backscattered electron detector (SE+QBSD); (**e**) groove 16 mW SEM, SE+QBSD; (**f**) groove 16 mW SEM, SE+QBSD at higher magnification; (**g**) groove 16 mW SEM, InLens at high magnification; (**h**) confocal z-stack imaging of the 16 mW sample. NPs: nanoparticles.

Figure 4 contains images of the investigated samples. Figure 4a presents the surface of the gold-coated PEEK sample after laser micromachining. The honeycomb pattern processing caused partial material ablation and remelting, which resulted in a periodic repetitive change of composition. The grooves presented in Figure 4b in higher magnification shows an undisturbed groove with gold particles remelted in the polymer matrix, highlighted using the light microscopy technique. Figure 4c–e were carried out using scanning electron microscopy (SEM) and a mix of secondary electron (SE) and Quadrant Backscatter Electron Detector (QBSD) techniques to present an even distribution of numerous gold nanoparticles in the polymer matrix for every sample preparation variant 4.5 mW, 15 mW and 16 mW, respectively. Additionally, in Figure 4f,g there are images of the 16 mW sample obtained using high magnification to show the particles size ≤10 nm. Figure 4h presents topography of the obtained single-pulse irradiated area.

**Figure 5:**
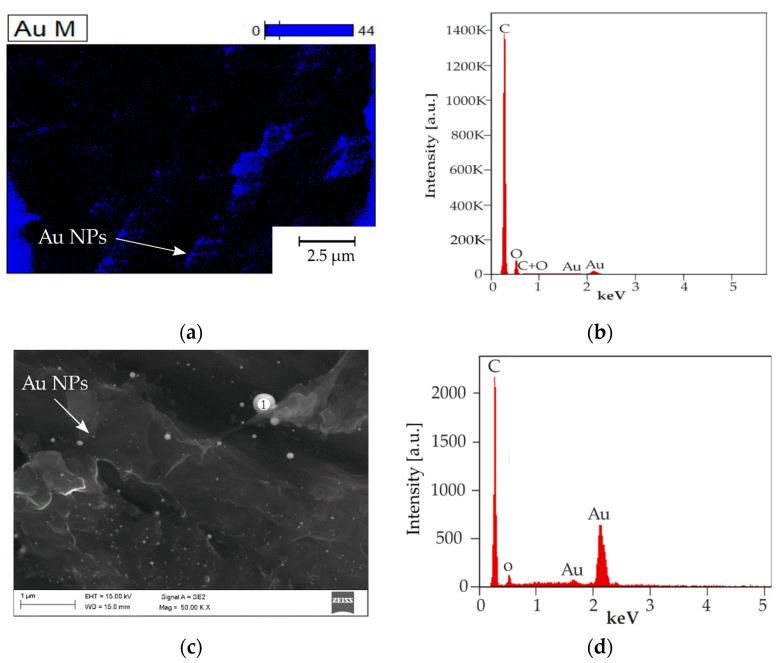
EDS qualitative chemical elements distribution map and X-ray energy dispersion spectrum: (**a**) Au distribution map—Au marked as blue; (**b**) EDS X-ray spectrum from Figure 5a area (**c**) SEM image with localisation of EDS measurement point 1 (**d**) EDS X-ray spectrum from point 1 from Figure 5c.

The obtained results were confirmed by energy dispersive spectrometry (EDS) presented on a chemical elements distribution map in Figure 5a and an X-ray energy dispersion spectrum in Figure 5b. The higher magnification measurement EDS point was obtained in Figure 5c with the spectrum presented in Figure 5d.

**Figure 6:**
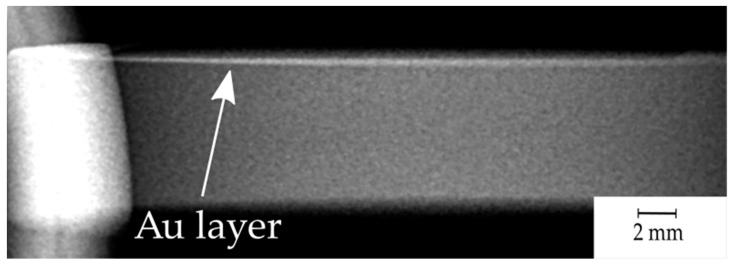
X-ray image (side view) of the sample with the revealed gold layer (top).

The visibility of the sputtered gold layer was also revealed by a medical X-ray system. Figure 6 presents a side view of the sample.

**Figure 7:**
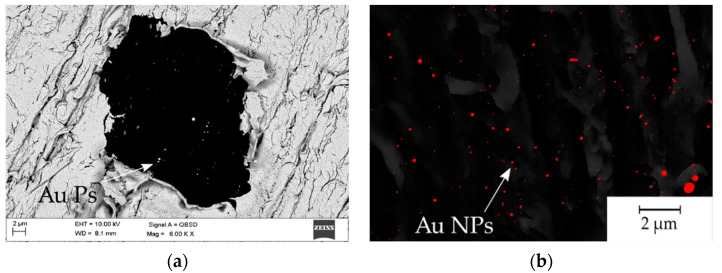
SEM images prepared for the statistical analysis of particle distribution: (**a**) 4.5 mW single laser irradiation spot, SEM, QBSD; (**b**) SEM, SE+QBSD image threshold to quantitative analysis of the Au particles.

**Figure 8:**
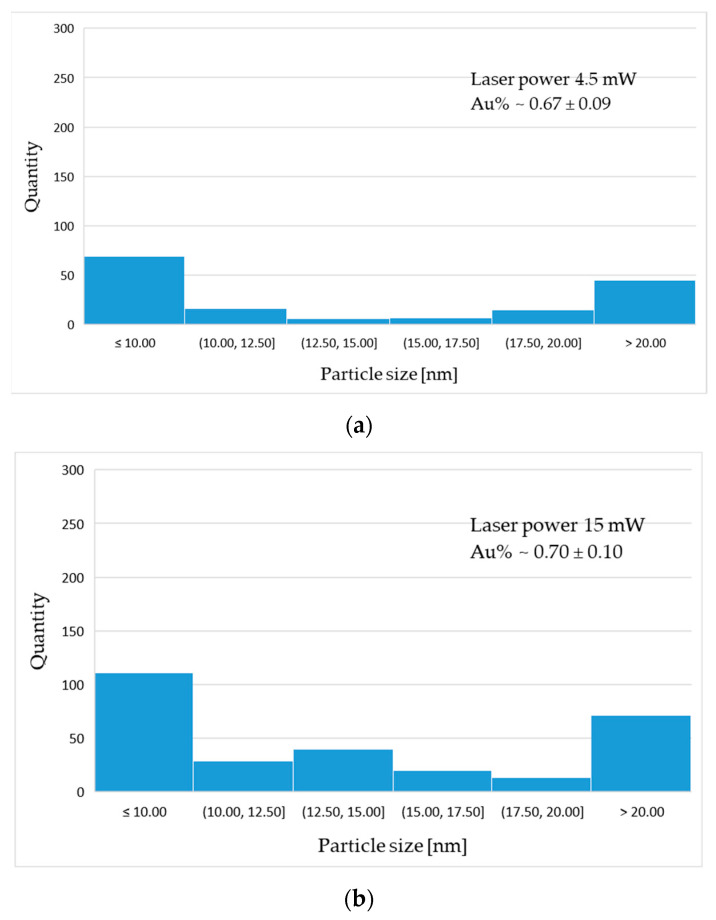

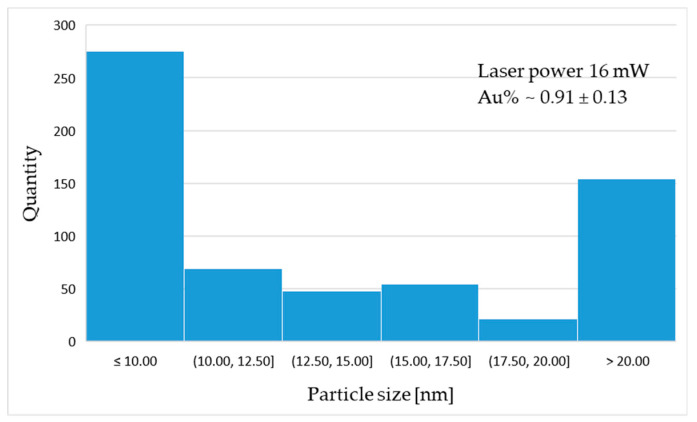
Histograms of particle distribution for sample: (**a**) 4 mW; (**b**) 15 mW; (**c**) 16 mW.

The gold particle distribution was determined based on high resolution SEM image analysis, shown in Figure 7. The statistical distribution of gold particles is presented in the form of histograms (Figure 8). The average percentage of Au distribution in the field of microscope view is 0.67; 0.70; 0.91% for samples 4.5 mW, 15 mW and 16 mW, respectively. The diagrams show the prevalence of nanoscaled particles which, regardless of the laser power, are distributed through the whole groove area. 

The statistical analysis of particle distribution indicates the presence of nanometric particles mostly 10 nm or less in diameter. As the laser power increases, the proportion and size of particles present in the remelted area changes.

## 4. Discussion

To assess the laser beam absorbance of the PEEK material in order to choose the best laser treatment parameters, taking into account final application properties, the experiment of laser power to groove depth correlation was carried out. Based on the current state-of-the-art results [2,33,34] one can state that for cardiovascular purposes, the depth of textured patterns should not exceed 5 µm. The following condition is motivated by the delicacy of the blood system implants’ geometry and the need to avoid thrombus formation. Nevertheless, the first trials of laser surface treatment in high frequency modes made the whole metallic material evaporate from the groove, leaving an almost clean surface of PEEK. Inasawa et al. [35] revealed that the size reduction of Au nanoparticles generally occurs by a layer-by-layer mechanism based on the bimodal distribution of particle sizes that are smaller with every next cycle of laser irradiation. They also indicated that among different impulsive lasers, the picosecond laser irradiation is the most efficient way to induce size reduction in the case of Au and Ag nanoparticles [35,36]. 

**Figure 9:**
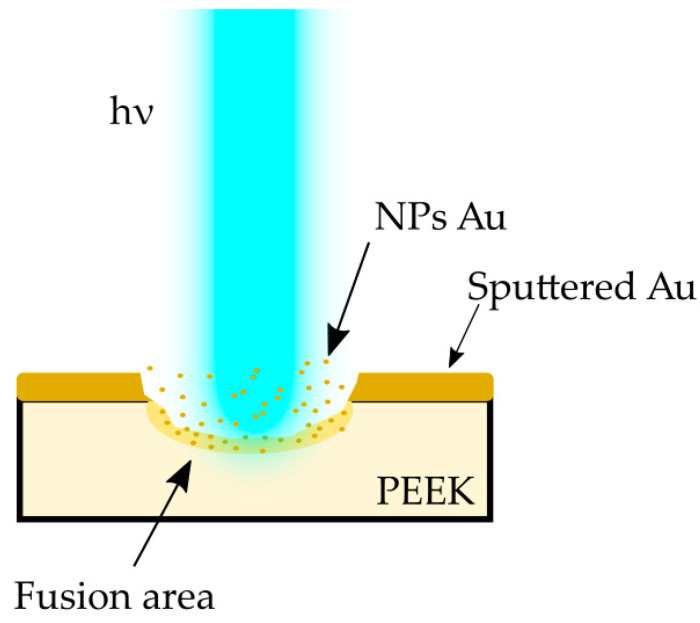
Scheme showing the interaction of a laser beam with matter.

In the case of our experiment, with the gold sputtered layer being approximately 200 nm thick, there was the need to stabilize the process of NP reduction by the single-pulse laser irradiation. Based on the research carried out by Pyatenko et al. [37], different mechanisms of particle size reduction apply under different experimental conditions. The processes caused by electron ejection depends on the laser energy flow density, which, in turn is a function of particle diameter and laser wavelength. Pyatenko et al. defined that if the energy flow density is less than 10^12^ W/m^2^, the particles reduce their size in a mechanism following the strict order of heating–melting–evaporation. This indicates that in the case of using a laser micromachining system, with the average energy flow density not exceeding 3.2 × 10^7^ W/m^2^, that particular mechanism will occur almost exclusively. Taking into account the specifics of the laser treatment processes, this contribution presents results of experiments aiming to achieve the effect of gold NP fusion in the polymer matrix. The fusion of gold nanoparticles into the PEEK polymer is illustrated in the Figure 9.

The light and SEM microscopy allow one to assess the presence, distribution and size of the obtained Au NPs in three different laser powers. The EDS spectra confirm the presence of gold, and the EDS map suggests a random homogenous particle distribution, depending on the topography of the polymer melt structure, but based on QBSD imaging this dependence was not visualised. The statistical analysis validated the size, distribution and quantity of particles and allowed one to assess the average percentage of Au to polymer ratio. In general, it can be observed that the higher the power of the laser beam, the percentage of particles present in remelted area changes. 

The obtained results show that the income of <10 and >20 nm sized particles is greater between the range of high laser energy (15 mW to 16 mW) than between 4.5 mW to 15 mW. This finding suggests that gold disintegrates with greater efficiency at higher laser powers. The average percentage of Au suggests that the higher the laser power, the more particles are present in the polymer matrix. The percentage content of Au NPs is shown in Table 4. This phenomenon is desirable due to a better distribution of bigger diameter particles across the surface. Additionally, such behaviour may increase antibacterial properties, improving biocompatibility. Similar experiments have been carried out before on PEEK with the use of Ag nanoparticles obtained by different methods [38]. Laser-assisted synthesis of Au nanoparticles has been described before in liquid (water, as the beam energy provider) to dissolute potassium tetrachloroaurate (III), KAuCl_4_ fractions. Aqueous [AuCl_4_]^−^ in this approach formed particles with a controlled nano-sized diameter by photochemical reduction [39]. Laser irradiation of chitosan films, with tetrachloroauric acid (HAuCl_4_) as a precursor to releasing Au NPs was described in [40]. Mentioned works have presented methods using [AuCl_4_]^−^ anions to produce NPs by the photochemical reaction (photo-fragmentation) and have turned out to be very promising. However, the truly physical method based on pure Au constituting NPs and laser surface treatment seems to be not sufficiently explored. The application of Au and Ti coatings in PEEK bioactive improvement strategies is noted [41,42,43], but there seems to be lack of extensive analysis of Au coating constitution conducted with laser-assisted particle size reduction approach. 

The idea of enriching the PEEK polymer with Au NPs by laser treatment is reasonable and promising, because of a high X-ray attenuation coefficient at the energy levels utilized for medical X-ray and computer tomography (CT) [22,44,45]. Even a small amount of Au should be visible through X-ray detection. The obtained X-ray image (Figure 6) shows a straight relatively sharp radiation glow line along the coated plane, which confirms the authors’ goals and allows precise implant localization, also allowing one to reveal potential degradation and its control. The absence of shading of gold in the X-ray image will indicate the wear of the implant, which, due to PEEK’s great chemical resistance, should last for a long time without any symptoms of wear [41,46]. Gold being highly biocompatible does not tend to cause any side effect of being released inside the body [23,24]. The process carried out to produce biomimetic patterns on the surface has also been reported in the literature and in numerous studies to be positive for body response and mechanical properties, the analysis of which will be included in future studies [3,5,34].

## 5. Conclusions

Preliminary research of laser-assisted deposition of gold nanoparticles in PEEK polymer allows for a positive assessment of the applied technique. After the research, the following conclusions were made:

- The Au NP fusion inside the PEEK matrix is possible for the proposed in experiment laser power.
- The more energy is applied to the micromachined area, the more average percentage of Au is presented in the lasered area.
- The influence on laser power on the effectiveness of reducing particle size during the process. The lower the power, the lower the number of particles observed.

Further analysis of the mechanical and surface properties of the material is required, as well as further optimisation of the process to increase the percentage of gold infusion of the polymer matrix.

## Figures and Tables

**Table 1:** PEEK properties; heat resistance and stability [29].

PEEK Properties	Value
Long-term heat resistance	260 °C
Short-term heat resistance	300 °C
Glass transition point	150 °C
Melting point	341 °C
Thermal deformation temperature HDT-A	162 °C
Flame resistance:	UL94 V0
Medical standard	ISO 10993 [30]

**Table 2:** Sample treatment and identification.

Name	Surface Treatment
4.5 mW	Polished; Au coated; laser treated with 4.5 mW
15 mW	Polished; Au coated; laser treated with 15 mW
16 mW	Polished; Au coated; laser treated with 16 mW

**Table 3:** Laser texturing process parameters.

Cutting/Etching Speed	Frequency	Laser Power	Beam Width	M^2^
-	-	4.5 mW	-	-
1 mm/s|10 mm/s	10 Hz|400 Hz	15 mW	~30 µm	<1.2
-	-	16 mW	-	-

**Table 4:** Average filling level of Au NPs.

Sample Name	Average Au% in Single Microscopic View
4.5 mW	0.67 ± 0.09
15 mW	0.70 ± 0.10
16 mW	0.91 ± 0.13

## Data Availability

Not applicable.
